# Efficacy of a Training on Executive Functions in Potentiating Rehabilitation Effects in Stroke Patients

**DOI:** 10.3390/brainsci11081002

**Published:** 2021-07-29

**Authors:** Vincenza Tarantino, Francesca Burgio, Roberta Toffano, Elena Rigon, Francesca Meneghello, Luca Weis, Antonino Vallesi

**Affiliations:** 1 Department of Psychology, Educational Science and Human Movement, University of Palermo, 90128 Palermo, Italy; 2 IRCCS San Camillo Hospital, 30126 Venice, Italy; francesca.burgio@ospedalesancamillo.net (F.B.); roberta.toffano@virgilio.it (R.T.); elena.rigon@ospedalesancamillo.net (E.R.); francesca.meneghello@ospedalesancamillo.net (F.M.); 3 Department of Neuroscience, University of Padova, 35128 Padova, Italy; luca.weis@unipd.it; 4 Department of Neuroscience & Padova Neuroscience Center, University of Padova, 35128 Padova, Italy

**Keywords:** executive functions, stroke patients, cognitive training, rehabilitation, brain lesion

## Abstract

Cognitive impairment after a stroke has a direct impact on patients’ disability. In particular, impairment of Executive Functions (EFs) interferes with re-adaptation to daily life. The aim of this study was to explore whether adding a computer-based training on EFs to an ordinary rehabilitation program, regardless of the specific brain damage and clinical impairment (motor, language, or cognitive), could improve rehabilitation outcomes in patients with stroke. An EF training was designed to have minimal motor and expressive language demands and to be applied to a wide range of clinical conditions. A total of 37 stroke patients were randomly assigned to two groups: a training group, which performed the EF training in addition to the ordinary rehabilitation program (treatment as usual), and a control group, which performed the ordinary rehabilitation exclusively. Both groups were assessed before and after the rehabilitation program on neuropsychological tests covering multiple cognitive domains, and on functional scales (Barthel index, Functional Independence Measure). The results showed that only patients who received the training improved their scores on the Attentional Matrices and Phonemic Fluency tests after the rehabilitation program. Moreover, they showed a greater functional improvement in the Barthel scale as well. These results suggest that combining an EF training with an ordinary rehabilitation program potentiates beneficial effects of the latter, especially in promoting independence in activities of daily living.

## 1. Introduction

Stroke is a cerebrovascular accident, due to ischemia or hemorrhage, which causes loss of brain function. This disorder is characterized by a fast and focal development of neurologic symptoms along with motor, language, and other cognitive impairments. According to the World Health Organization, stroke is the second leading cause of death and the third leading cause of disability [1]. More than two-thirds of stroke survivors have limitations to live independently or have catastrophic impact in their daily life. Cognitive dysfunctions affect more than one third of these patients, may persist for years after stroke, and strongly contribute to disability. Prospective studies have shown that the cognitive status is a key factor in post-stroke recovery [2,3]. Patients with cognitive deficits at three months are 2.4 times more likely to have dependent living, irrespective of age and severity of physical impairment [4]. Given these prognostic implications, an adequate and timely cognitive assessment, together with an appropriate rehabilitative intervention, are of paramount importance.

Impairment of executive functions (EFs) represents one of the most common cognitive sequelae of stroke. Depending on the definition and instruments used for its assessment, the prevalence of executive dysfunction after stroke ranges from 19% to 75% [5]. In spite of this, some of them are ‘invisible’ to the patient’s awareness. Namely, while motor, language, and memory impairments are more apparent, deficits involving some EFs (such as working memory, sustained and shifting attention) are more insidious or manifest when patients fully return to the complex demands of daily life [6]. Therefore, even the treatment plans risk neglecting them.

EFs refer to high-level cognitive operations that direct lower-level processes toward goal-oriented actions [7]. This class of cognitive functions encompasses multiple abilities, including planning, problem solving, criterion setting, multitasking, working memory, cognitive control, inhibition, switching, and monitoring. All these functions are strongly interrelated but also separable and independent [8,9]. The complexity of EFs makes them very sensitive to brain changes resulting from stroke [10]. EF impairment is expected to generate cascade effects on other cognitive functions and to reduce the capacity to regain independence in activities of daily living [11]. Furthermore, it affects the ability to adapt to new or problematic situations, such as when alternative movement strategies are necessary to compensate for limb weakness or when planning in advance a sentence is required for rehabilitating verbal communication. Therefore, executive dysfunctions greatly affect the quality of a patient’s life and self-sufficiency, resulting in everyday difficulties across diverse neurological conditions [12,13,14].

Studies that investigated the role of executive functioning in recovery after stroke have found that this is a strong predictor for recovery of motor functions as well as dependence in daily living after discharge [15,16,17]. Previous systematic reviews on EF rehabilitation in stroke [5,18,19] concluded that high-quality evidence about the effect of EF rehabilitation is still limited but encouraging. In particular, computer-based rehabilitation programs, which are being increasingly used in clinical practice, are promising, although their effect on the improvement of cognitive functions after stroke and, more importantly, in activities of daily living, still needs further evidence [19]. Of note, some studies found benefits of using a combined approach to cognitive rehabilitation in brain injury, that is, to use computer-based rehabilitation programs in addition to conventional programs [20].

The goal of the present study was to evaluate the efficacy of a computerized training program aimed at potentiating EFs in patients with stroke. The peculiarity of the program is that it was aimed to be combined with ordinary rehabilitation programs (on speech, language, motor functions, etc.) and that its tasks demand minimal selective attention, expressive language, and motor resources. As such, it could be administered to a wide range of patients, regardless of the specific brain damage and deficits, even to those patients with severe impairments but preserved basic motor (movement of one hand) and language (comprehension of simple sentences) functions. The underlying hypothesis was that if EFs regulate more basic functions, from motor to more cognitive ones, then, strengthening EFs would support the recovery of these functions. Therefore, the combination of this EF training program with an ordinary rehabilitation, which could involve conventional as well as computerized interventions, both tailored to the patient (such as cognitive rehabilitation on specific attention, memory, and EFs), would improve final rehabilitation outcomes, especially daily life functions.

To this end, a training group and a well-matched control group were tested at two time points, before (T0) and after (T1) rehabilitation. The training group performed a computerized training to potentiate EF, in addition to the standard rehabilitation program (i.e., treatment as usual). The training was designed based on a brain-based EF model inspired by the ROBBIA model [9,21,22,23], and was adaptive. The control group underwent the standard rehabilitation program only. To evaluate the efficacy of the training program on the final rehabilitation outcomes, patients’ performance on a battery of standardized tests covering multiple cognitive domains, and scores on functional scales were measured at T0 and T1. We hypothesized a greater improvement in cognitive functions and in independence in daily life in the group that underwent the EF training compared to the control group.

## 2. Materials and Methods

### 2.1. Procedure

The study was conducted at the IRCCS San Camillo Hospital, Venice (Italy), a specialized rehabilitation hospital. The protocol was conducted in compliance with the Declaration of Helsinki and received formal approval by the local Ethical Committee. All patients signed an informed consent prior to their participation. Exclusion criteria were: age below 18 years, premorbid ischemic or hemorrhagic stroke, comorbid neurological (e.g., epilepsy) or neurodegenerative (e.g., multiple sclerosis, dementia) disorder, premorbid or comorbid brain damage other than stroke (e.g., traumatic brain injury, tumor), psychiatric disorders, unstable medical conditions, sensory impairments that prevent processing and recognition of visual stimuli (e.g., agnosia or color blindness), dyslexia/alexia, severe vigilance or verbal comprehension deficits, impaired motor control of both hands. Patients were enrolled in the study during their hospitalization and were randomly assigned to one of the two groups. A single-blind randomized controlled design was adopted and a stratified randomization procedure guaranteed a balance of the two groups in terms of age, sex, education, time from event, and stroke severity, as assessed by the neurological examination. All participants received an inpatient rehabilitation program, based on their needs, in accordance with the routines at the clinic. The rehabilitation program could include: neuromotor rehabilitation (e.g., robotic gait training, virtual reality, and cycling), speech rehabilitation (e.g., conversational therapy, reading, and writing), occupational therapy (daily living activities, group activities, and garden therapy activities that engage patient in all stages of plant cultivation and care), and optokinetic stimulation, but also neuropsychological rehabilitation on specific cognitive abilities, such as visuo-spatial exploration, sustained attention, working memory, problem solving, planning, and performance monitoring. The typical rehabilitation program for stroke patients usually lasts from two to three months.

In addition to the rehabilitation program, the training group received the EF training as well. The training lasted 10 sessions, about one hour each, distributed over a mean of 15.7 days ± 2.3 SD. Both groups were assessed with a battery of neuropsychological tests at T0 (before training and rehabilitation or rehabilitation only), and at T1 (after training and rehabilitation or rehabilitation only). The time interval between T0 to T1 lasted about 40 days (45 days ± 19.5 for the training group, 40 days ± 16.3 for the control group, *t*(35) = 1.14, *p* = 0.439). Furthermore, measures on independency in activities of daily living were assessed at hospital admission and at hospital discharge (3.5 months ± 1.3 for the training group, 4.2 months ± 2 for the control group, *t*(35) = 1.22, *p* = 0.229), by means of functional scales.

### 2.2. Participants

Patients were consecutively selected according to their hospital admission, scrutinized according to the exclusion criteria, and then approached to ask for participation. A total of 43 patients, admitted from January 2018 through August 2020, were enrolled. Three patients were lost to follow-up assessment (T1), whereas three patients discontinued rehabilitation (see Figure S1 for a detailed patient flow). The final sample included 18 patients in the training group and 19 patients in the control group. Patients’ characteristics, namely age, sex, education, and Mini-Mental State Examination (MMSE) standardized score [24]; TIB standardized score (Italian version of the National Adult Reading Test [25]), etiology (ischemic or hemorrhagic), time since event, symptoms reported at the hospital admission, and the rehabilitation programs completed during hospitalization are summarized in Table 1. Two participants were left-handed (one in the training group, one in the control group), and one was ambidextrous (in the training group). The a priori power calculation had estimated a sample of 16 patients per group in order to detect a small effect of training (partial *η*^2^ = 0.04), with a statistical power (1 − *β*) of 0.80, a significance level (*α*) of 0.05, and a repeated-measures correlation of 0.7 (G*Power 3 software [26]).

No significant differences at baseline were observed between groups with regard to age (*t*(35) = 0.068, *p* = 0.946), sex (χ^2^ = 0.218, *p* = 0.728), education (*t*(35) = 0.094, *p* = 0.925), MMSE (*t*(31) = 0.512, *p* = 0.613), TIB (*t*(31) = 1.342, *p* = 0.189), time since event (*t*(35) = 1.14, *p* = 0.259), etiology (χ^2^ = 0.013, *p* = 0.999), lesion side (χ^2^ = 0.09, *p* = 0.687), symptomatology (χ^2^ = 1.55, *p* = 0.671), and type of rehabilitation (χ^2^ = 0.992, *p* = 0.803).

**Figure 1:**
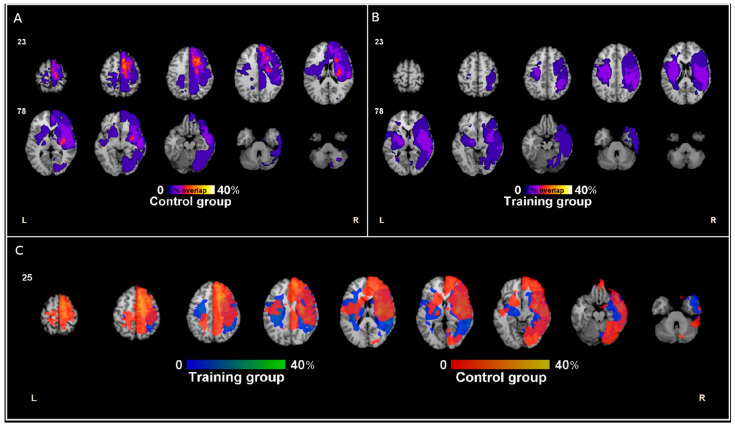
Lesion coverage proportion maps of training and control groups. Lesions were manually segmented and overlaid onto the reference standard brain template by means of the clinical toolbox [27,28]. The percentage values represent the lesion overlap within (**A**,**B**) and between (**C**) groups.

Structural information of brain lesions was obtained from regular MRI (T1-weighted, T2-weighted and/or FLAIR scans) or computed tomography (CT). Figure 1 describes the overlap of patients’ lesions (two patients were excluded from this lesion overlap image due to poor data quality).

### 2.3. Training Program

The rationale of the program is partially founded on studies conducted in our laboratory and previously in the Don Stuss’ lab, which elaborated a brain-centered model of EFs based on two distinct domain-general EFs, namely, criterion setting and monitoring [9,21,22,23]. In line with this model, the program included four types of training tasks, targeting Working Memory (WM), Interference Control and Inhibition (ICI), Task-Switching (TS), and Monitoring (M). In each task, stimuli consisted of “cards”, displayed one at a time in the center of a laptop screen. The cards could contain one of the following stimuli: geometric symbols, words, faces, and objects. Since the training was aimed at potentiating domain-general processes, regardless of the specific materials, the type of stimuli varied within and across sessions. The order of the sessions/stimuli was counterbalanced across patients. The tasks were presented in a fixed order (WM, ICI, TS, and M), following a hierarchical logic. This logic relies on a hierarchical integrative model [29], based on developmental evidence, according to which working memory is the component that develops first, followed by inhibitory control and, finally, cognitive flexibility, which is built on both of them. The duration of the stimulus presentation was adapted to the single patient’s performance. Each task lasted approximately 10 min. All tasks were designed to involve minimal load on selective attention, expressive language, and motor abilities. Namely, the cards were displayed one at a time on the screen, and in all tasks the response required only a button press of the spacebar, with the index finger of the dominant hand (or the index finger of the non-hemiplegic hand), without selecting among alternative keys. Patients were unaware of all task manipulations. Stimulus presentation and data recording were controlled by the E-Prime 2 software (Psychology Software Tools, Pittsburgh). Each patient was tested individually in a quiet and dedicated hospital room, with the continuous presence of a junior neuropsychologist (R.T. or E.R.), who set up the laptop and assisted the patient with the instructions.

#### 2.3.1. Stimuli

Geometric symbols were adapted from a popular US game (www.setgame.com, accessed on 28 July 2021) and could differ in shape (ovals, diamonds, or flags), color (red, blue, or green), or number (from one to three). Faces could be female or male, Caucasian or not, neutral or emotional (adapted from [30]). Objects could be kitchen or garage tools, unimanual or bimanual, and made with metal or not (adapted from [31]). Words could refer to animals, fruits, or vegetables and begin with a specific letter. Examples of stimuli and trials are reported in Supplementary Materials.

#### 2.3.2. Working Memory (WM) Task

The WM task was based on the 1-back paradigm [32]. Patients had to respond by pressing the spacebar whenever the card was identical to the one presented in the immediately preceding trial. A simple response time procedure was chosen, instead of the typical two-choice procedure, in order to unload the selection process.

The task comprised five blocks of 20 trials each (seven targets). The first two blocks were practice blocks and served to make patients familiarize with the task. During these blocks, feedback on each trial was provided to ensure instruction understanding. In the first block, the cards were displayed for 1500 ms and followed by a blank screen for 3000 ms (Inter-Trial-Interval, ITI). If the patient reached an accuracy >65%, then the stimulus duration and ITI were increased on the successive blocks to 1000 and 2500 ms, respectively. If the accuracy level was <65%, then the practice block was repeated until an accuracy level >65% was reached. If the patient obtained an accuracy level >85% in three consecutive sessions, a duration manipulation was applied, namely, the ITI duration was lengthened to load the process of stimuli retention in memory (to 3500, 4000, or 4500 ms).

#### 2.3.3. Interference Control and Inhibition (ICI) Task

This task was designed to train both “cognitive” and “motor” inhibitory processes. Again, a series of cards was presented, one at a time, centered on the screen. Participants were required to respond whenever a card belonging to a specific category appeared (targets) and not to respond to the other ones. For example, in the case of geometric symbols, patients were instructed to respond to all red diamonds. In this case, the targets were cards containing one, two, or three red diamonds only. Cards with red ovals, red flags, green diamonds, and blue diamonds, represented distractor stimuli, since they share some but not all features with the target (i.e., color and shape, respectively). All other cards represented simple no-go stimuli. Alternatively, in the case of faces, participants could be required to respond to happy female faces. In this case, the targets were happy Caucasian and non-Caucasian female faces. Happy male faces and neutral female faces represented distractor stimuli, whereas neutral and sad male faces represented no-go stimuli. Withholding response to distractor trials required interference control, whereas withholding response to non-go trials required more general inhibition.

The ICI task comprised four blocks. The first two blocks were for practice and contained 18 trials each. According to performance levels (< or >65%) on practice blocks, the stimulus duration was set to 1500 or 1000 ms, and the ITI to 3000 or 2000 ms. The two successive task blocks had two different target types and contained 64 trials each (16 targets).

#### 2.3.4. Task-Switching (TS)

Patients were asked to perform the same task as the ICI, namely to detect a target card. Yet, unlike the ICI task, shorter series of trials (9, 12, or 15) were presented. At the beginning of each series, the target card was displayed, which was one of the two target types used in the ICI task. For each series, the target card could be the same as the preceding series (“repeat series”) or could change (“switch series”). Compared to repeat series, in switch series participants were required to disengage from the previous/alternative target. Therefore, commission errors (i.e., responses to cards containing the alternative target) would reflect a failure of this process. The target cards varied across sessions.

#### 2.3.5. Monitoring Task (M)

As in the TS task, short series of cards (9, 12, or 15) were presented one at a time, for 1500 ms, in the center of the screen. At the beginning of each series, a target card was shown. Patients had to detect as soon as possible this card among the series, which appeared towards the end (at the 8th, 9th, or 14th trial). In “predictable” series, the presented card followed a certain regularity (such as increasing number of symbols), which allowed the anticipation of the target occurrence. In “unpredictable” series, the cards presented in the series were randomly chosen. The M task trained the ability to monitor events in order to, implicitly, check rules (e.g., [33]). Two practice series were presented at the beginning, to familiarize with the instructions. Then, six predictable series and six unpredictable ones were administered. The patient was not aware of the predictability manipulation. 

### 2.4. Outcome Measures

Two types of outcome measures were analyzed to quantify the effect of training: performance on neuropsychological tests and scores on functional scales. The neuropsychological test battery assessed short-term and working memory (Digit span forward and backward, Corsi block-tapping test [34]); attention and processing speed (Attentional matrices [35], Trail Making Test–A [36]); language (Naming test [37], Phonemic and Semantic fluency [38]); and executive functions (Wisconsin Card Sorting Test, WCST [39,40], Five Point test [41,42], and Stroop test [43]). Some composite scores were considered: the error index in the Five Point test, which measures the percentage of perseverative or rule-breaking errors over the total number of designs [41], and the Stroop Inverse Efficiency Score (IES), which is derived by dividing mean Stroop interference response time by its corresponding accuracy. Additionally, the patients were tested with the Barthel Index [44] and the Functional Independence Measure (FIM^TM^ [45,46]). These scales evaluate the level of disability in everyday contexts, namely, the dependency on a caregiver. The Barthel index allows the assessment of changes in the basic daily activities (such as self-care and locomotion) and consists of 10 items, scored on a five-point Likert scale, with total score ranging from 0 (totally dependent) to 100 (totally independent). The FIM assesses physical and cognitive changes in daily contexts (such as self-care and communication abilities) and consists of 18 items, scored on a seven-point Likert scale, with total score ranging from 18 (totally dependent) to 126 (totally independent). Disability severity did not differ between the two groups at T0, as indexed by the Barthel (training group: range 0–70, control group: range 0–65; *t*(35) = 0.156, *p* = 0.877) and the FIM score (training group: range 23–82, control group: range 24–83; *t*(35) = 0.533, *p* = 0.597) score.

### 2.5. Data Analyses

Statistical analyses were conducted using Statistical Package for the Social Sciences (SPSS) version 22.0 (IBM SPSS Statistics 2014). All dependent measures, namely, all tests and scales’ scores, were continuous variables. According to the Kolmogorov–Smirnov test, some neuropsychological measures were normally distributed (TMT-A, Attentional matrices, Phonemic fluency, Semantic fluency, Five Point error index, and Barthel index), whereas some other measures were not (i.e., Digit span forward, Digit span backward, Corsi block-tapping test, Naming, WCST categories, WCST errors, and Stroop IES, FIM). In order to examine significant difference in scores between T0 and T1, paired *t*-tests, or Wilcoxon signed-rank test for the not normally distributed variables, were computed, separately for each group. To test group differences in Pre vs. Post changes, an ANCOVA model was applied to normally distributed variables, which included the score at T1 as a dependent variable, and the score at T0 as covariate (Post ~ Pre + Group + Pre × Group). This is considered the best analytical approach for examining data from two-group pre-post designs [47,48]. If, on the one hand, it adjusts for pre-treatment measures, it also takes into account that baseline individual scores affect cognitive training effects, namely, a negative relationship between a participant’s initial cognitive ability and the results of training [49,50]. For testing not normally distributed measures, the ANCOVA was conducted on rank-transformed scores [51,52]. The effect sizes for pre-test –post-test control group designs were estimated, based on the mean pre–post change in the treatment group minus the mean pre–post change in the control group, divided by the pooled pre-test standard deviation (d_ppc2_ [53]). *p*-values < 0.05 for a two-sided test were considered statistically significant.

## 3. Results

Table 2 contains the mean scores (and standard deviations) for each test and group, at T0 and T1. The paired *t*-tests showed significant improvement at T1 relative to T0 for the Attentional matrices (*t*(17) = 4.23, *p* = 0.001) and for the Phonemic fluency (*t*(13) = 2.64, *p* = 0.020) scores, in the training group only. A tendency toward significance was also found in the Digit Span forward score (*Z* = 1.96, *p* = 0.05). Interestingly, a significantly higher proportion of patients in the training group shifted their performance to the normal range in the Attentional matrices test at T1 relative to T0 (McNemar test = 4.2, *p* = 0.031).

Scores on the functional scales revealed significant improvements from the hospital admission to the discharge for both groups (Barthel index: training group *t*(17) = 8.83, *p* < 0.001; control group *t*(18) = 9.93, *p* < 0.001; and FIM: training group *t*(17) = 9.8, *p* < 001, control group *t*(18) = 8, *p* < 0.001). All patients, except one patient in the training group and one patient in the control group, underwent an improvement in the Barthel index above the minimal detectable change (MDC) expected in test–retest assessment [54].

In Table 2, the group effect size on the post–pre comparison was reported. This pre-test–post-test control group size effect was larger than 0.4 in the Digit span, Phonemic fluency, errors on the WCST, Stroop interference (IES), and, more importantly, in the Barthel index. In all these measures, the ordinary rehabilitation combined with the training yielded a larger effect than the ordinary rehabilitation only. Namely, performance at T1 relative to T0 improved more in the training group relative to the control group in all tests. Only the Naming accuracy showed a larger positive change in the control group.

**Figure 2:**
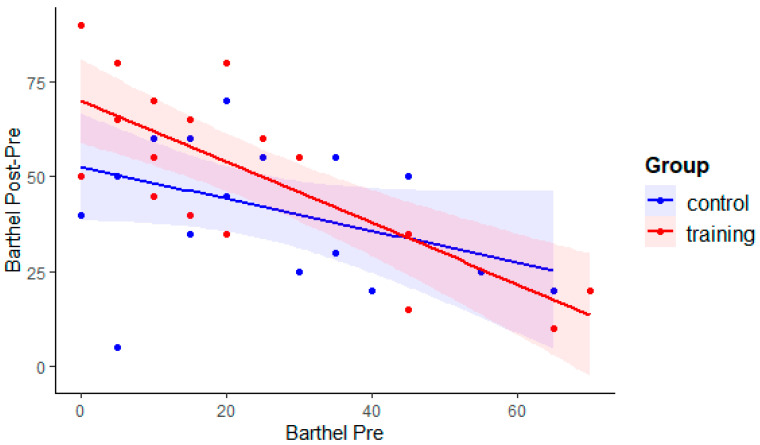
Relation between scores on the Barthel Index before rehabilitation (Pre) and the difference scores between Post- and Pre-rehabilitation, in the training and control groups.

The ANCOVA model revealed that, when controlling for T0 scores, a significant Group effect emerged on the post-rehabilitation scores on the Barthel scale (*F*(1,33) = 4.2, *p* = 0.049, and partial *η*^2^ = 0.113). Namely, participants of the training group obtained higher scores at T1 compared to the control group. In order to explore the association between pre-rehabilitation Barthel scores and post–pre difference (gain) in Barthel scores, an ANCOVA with gain as dependent variable, pre-rehabilitation Barthel score as covariate, and Group as independent variable was performed. The results (see Figure 2) revealed a negative correlation between gain and pre-rehabilitation Barthel score (main effect of pre-rehabilitation Barthel score: *F*(1,33) = 21.32, *p* < 0.001, and partial *η*^2^ = 0.392), and confirmed an overall higher difference in the training group (*F*(1,33) = 4.2, *p* = 0.049, and partial *η*^2^ = 0.113). The interaction was not significant (*F*(1,33) = 2.12, *p* = 0.15), meaning that in both group pre-rehabilitation Barthel score and gain were correlated. As evident in Figure 2, patients with lower Barthel scores at T0 gained larger benefits than patients with higher Barthel scores at T0.

## 4. Discussion

The aim of this study was to investigate whether a computerized EF training could boost the effects of stroke rehabilitation when added to an ordinary rehabilitation program, regardless the specific brain damage and cognitive/motor impairments. To this aim, two groups of patients with stroke were involved in the study, a group that received the training in addition to the routine rehabilitation, and a group that received the routine rehabilitation exclusively. A neuropsychological assessment before (T0) and after (T1) the rehabilitation was administered to both groups (it is noteworthy that the same amount of time from T0 to T1 passed for the two groups). Furthermore, the self-sufficiency in the daily activities was assessed by means of two functional scales.

The findings showed that only the patients’ group that received the training improved performance on the Attentional matrices and on the Phonemic fluency test, at T1 relative to T0. The effect sizes computed for pre-test–post-test control group designs revealed a moderate effect of training in Digit span, Phonemic fluency, errors on the WCST, and Stroop interference. Both groups significantly improved in their independency in activities of daily living after rehabilitation, as measured by the Barthel and FIM scales. Remarkably, the training group obtained significantly larger improvement on the Barthel scale compared to the control group. This result suggests that the training boosted the effects of routine rehabilitation in the level of independence in activities of daily living. Although the FIM scale was expected to be more sensitive than the Barthel scale, in fact our results are consistent with previous studies that compared the two scales and found that the Barthel scale has good responsiveness in detecting changes after rehabilitation in patients with stroke [55].

The results on neuropsychological outcomes revealed that the training could improve functions not directly targeted by the training, such as visual selective attention (Attentional matrices) and verbal fluency (Phonemic fluency). This transfer effect to “near” cognitive functions is in line with the study hypotheses, and confirms previous evidence on the near transfer effects of EF trainings in healthy individuals [56] as well as in mild cognitive impairment [57] and brain-injured patients [58]. Moreover, the effects of the training on abilities not directly targeted by the training, such as on activities of daily living, provides support to the idea that the training of EFs might support the improvement of other “far” abilities (e.g., [59]).

As in this study, a previous randomized controlled trial has examined the effect of adding a computer-based training of working memory to routine rehabilitation programs in patients with working memory deficit following brain damage (e.g., stroke or trauma), and has shown greater improvements in the training group compared to the control one, not only in cognitive tests but also in Hospital Anxiety and Depression Scale scores [60]. Moreover, previous evidence documented the effectiveness of non-computer-based EF trainings (e.g., the Goal Management Training) in improving daily life activities when applied to patients with executive dysfunctions after various acquired brain injuries [61,62]. Unlike these investigations, the present work did not limit the training to patients with EF impairments and considered post-stroke patients only. We identified very few similar studies in the literature (summarized in Table S1), which combined a computerized attention and EF training with conventional rehabilitation in stroke patients, not specifically impaired on EF. Only two of them, out of five, found larger improvements in the trained compared to the control group on non-cognitive aspects, that is, higher patients’ satisfaction with the results of treatment [63] and lower anxiety and depression symptoms [64]. None of them, however, could observe greater improvement in daily living activities.

The improvements observed in this study might be attributed to some methodological strengths and novelties of the training. Namely, (i) it relied on minimal motor, language and attention requirements, therefore, all patients, even with multiple and/or severe impairments, could benefit from it; (ii) it was inspired by a brain-based model [9,21]; and (iii) it followed a hierarchical structure. Given the close correlation between EF changes and executive control fronto–parietal brain networks [65,66], we might speculate that the training had acted by reinforcing these networks (see [67]), although further replication with neuroimaging extension is desirable to confirm this hypothesis.

In order to test the robustness of the present findings and the maintenance of its effect over time, there is a need for further investigations adopting larger samples, a quantitative severity index, a more symmetric distribution of lesion’s side across patients, and a long-term follow-up. Furthermore, in order to maximize the likelihood of meaningful and stable gain and to obtain a larger effect size, the effect of additional training sessions should be assessed [68].

## 5. Conclusions

In conclusion, although these results are preliminary given the relatively low sample sizes, they suggest that adaptive computerized trainings on EFs added to conventional rehabilitation treatment, regardless of the specific stroke lesions and impairments, might be effective in potentiating/promoting recovery not only of related cognitive processes but also of broader functional abilities. We summarize the main contribution of this work in the finding that boosting EFs in an adaptive and theory-grounded manner might optimize functional recovery in post-stroke patients, even in cases where EF disorders are not specifically present. This training approach could potentially be applied to a wide range of clinical conditions, although the patients’ characteristics that would benefit the most from it should be investigated in future studies.

## Figures and Tables

**Table 1:** Demographic characteristics of the two patients’ groups. Mean (and standard deviation) or frequencies are reported in cells. MMSE: Mini-Mental State Examination; TIB: Italian version of the National Adult Reading Test. P-values were obtained from independent sample *t*-tests (Age, Education, MMSE, TIB, and Time since event) or chi-square (M/F, Etiology, Symptomatology, and Rehabilitation program) statistics.

	Training Group (*n* = 18)	Control Group (*n* = 19)	*p*
Age (years)	64.6 (12.7)	64.9 (12.7)	0.946
M/F	12/6	14/5	0.728
Education (years)	9.4 (4)	9.3 (4.2)	0.925
MMSE	25.1 (2.5)	24.6 (3.3)	0.557
TIB	106.1 (9.4)	101.1 (11.7)	0.189
Time since event (months)	3.1 (2.4)	4.2 (3.4)	0.259
Etiology			
Ischemia	13	12	0.999
Hemorrhage	6	6	
Lesion side			
Left hemisphere	7	7	0.956
Right hemisphere	9	11	
Bilateral	1	1	
Symptomatology			
Aphasia, dysartria	6	10	0.671
Neglect	7	8	
Sensory-motor impairments (e.g., hemiplegia/ hemiparesis)	18	19	
Cortical visual impairments (e.g., hemianopsia)	4	2	
Rehabilitation program			
Speech therapy	5	9	0.803
Motor therapy	18	18	
Occupational therapy	8	8	
Neuropsychological rehab	12	17	

**Table 2:** Means and standard deviations (in parentheses) for Pre- (T0) and Post-rehabilitation (T1) sessions. *P*-values derived from Wilcoxon signed-rank tests for not normally distributed variables (Digit span forward, Digit span backward, Corsi block-tapping test, Naming, Wisconsin Card Sorting Test WCST categories and errors, Stroop Inverse Efficiency Score, and Functional Independence Measure), and from paired *t*-tests for normally distributed variables (Trail Making Test-A, Attentional matrices, Phonemic fluency, Semantic fluency, Five Point error index, and Barthel index). The pre-test–post-test control group effect sizes were computed by the Morris’s formula [53]. In bold *p*-values < 0.050 and effect sizes > 0.4. N varies across tests because some participants could not perform all tests due to aphasia or neglect.

	Training Group				Control Group				
	T0	T1	*n*	*p*	T0	T1	*n*	*p*	*d*
Memory									
Digit span forward	4.6 (1)	5.2 (0.9)	16	0.050	4.7 (1)	4.9 (0.9)	16	0.331	**0.56**
Digit span backward	3.5 (0.8)	3.2 (1.1)	16	0.462	3.1 (1.3)	2.7 (1)	16	0.353	0.12
Corsi block-tapping	3.9 (0.9)	4.2 (1)	18	0.19	3.8 (1.1)	4 (0.8)	18	0.589	0.13
Attention and processing speed									
Attentional matrices	36.7 (13.5)	42.6 (12.7)	18	0.001	34.3 (10.6)	37.1 (10.2)	19	0.115	0.32
TMT-A (s)	87.1 (46.3)	82.3 (55.3)	17	0.543	77.9 (37.9)	68.9 (28.2)	16	0.077	0.16
Language									
Naming	14 (1.4)	14.1 (1.6)	17	0.276	13.8 (1.5)	14.3 (1)	17	0.069	−0.37
Phonemic fluency	24.1 (11.8)	29.1 (11.2)	14	0.020	21.6 (11.3)	22.6 (11.7)	16	0.569	**0.41**
Semantic fluency	33.8 (13.3)	34.2 (10.7)	9	0.872	25.7 (11.3)	26.6 (8)	15	0.554	−0.05
Executive functions									
WCST cat	4 (2)	4.2 (1.9)	16	0.521	3.2 (2)	3.2 (1.9)	16	0.807	0.13
WCST err	6.6 (4.4)	5.4 (6.3)	16	0.504	7 (4.7)	7.2 (5.1)	16	0.574	**−** **0.41**
Five Point error index	24.3 (22.1)	22.5 (24.3)	18	0.802	24.4 (20.3)	18.6 (12.8)	18	0.158	0.25
Stroop IES	45.2 (45.1)	37.5 (15.6)	15	0.532	47.7 (26.4)	44.6 (28.2)	13	0.158	**−** **0.49**
Functional scales									
Barthel index	25.5 (23.6)	75 (15)	18	<0.001	24.5 (18.5)	66.8 (20)	19	<0.001	**0.42**
FIM	56.3 (16.7)	92.8 (13.5)	18	<0.001	53.4 (16.4)	87.4 (21.1)	19	<0.001	0.19

## Data Availability

The data presented in this study are available on request from the corresponding authors.

## Supplementary Materials

The following resources are available online at https://www.mdpi.com/article/10.3390/brainsci11081002/s1, Figure S1: Consort flow diagram; Figure S2: Working Memory (WM) task, Figure S3: Interference Control and Inhibition (ICI) task, Figure S4: Task-Switching (TS), and Figure S5: Monitoring task (M); Table S1.
