# Supplementary material for: Efficacy of a Training on Executive Functions in Potentiating Rehabilitation Effects in Stroke Patients
Source: Brain Sci. 2021 Jul 29;11(8):1002. doi: 10.3390/brainsci11081002 (PMC8392264; doi:10.3390/brainsci11081002)
Supplement: Supplementary file 1 [file brainsci-11-01002-s001.zip › brainsci-1239483-supplementary.pdf]

Efficacy of a training on executive functions in potentiating rehabilitation effects in stroke patients – Supplementary materials

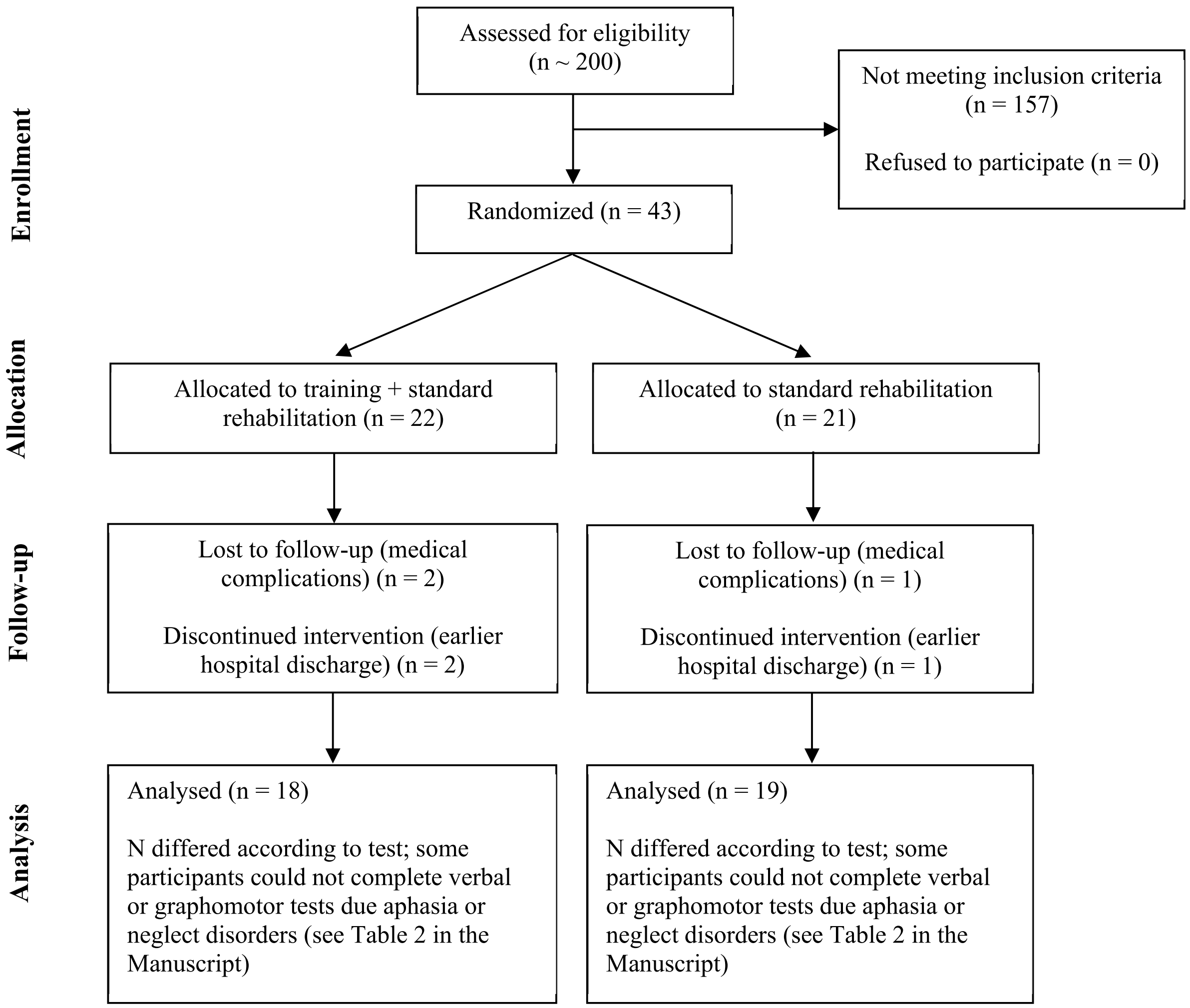

Figure S1. CONSORT flow diagram [1]. An approximate number of 200 patients were scrutinized to check for selection criteria.

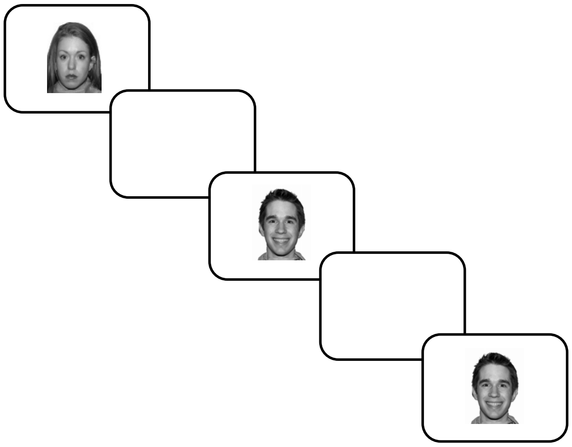

Figure S2. Working Memory (WM) task. Patients had to respond by pressing the spacebar whenever the displayed face was identical to the one presented in the immediately preceding trial. The timing of stimuli and blank duration was variable (see the Manuscript). Images were 230 × 300 pixels large and participants were positioned at about 50 cm distance from the laptop screen. The same task was presented with different stimuli across sessions (i.e., geometric symbols, words, or objects).

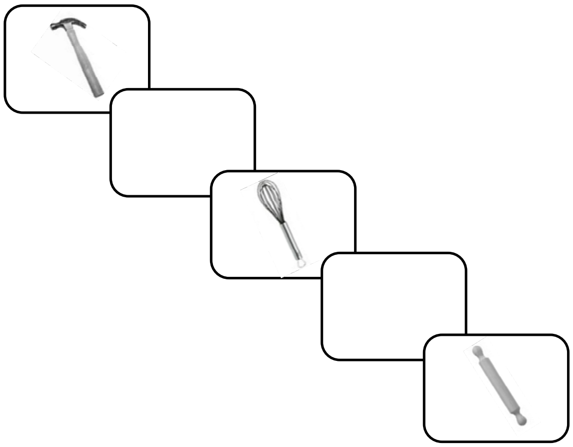

Figure S3. Interference Control and Inhibition (ICI) task. In the first block, patients had to respond by pressing the spacebar whenever a wooden kitchen tool appears. Therefore, working tools represent no-go trials, whereas non-wooden kitchen tools represent distracter trials. In the secondo block, metal working tools were the targets. The timing of stimuli and blank duration was variable (see the Manuscript). Images were contained in a rectangle 300 × 300 pixels and participants were positioned at about 50 cm distance from the screen. The same task was presented with different stimuli across sessions (i.e., geometric symbols, faces, or words).

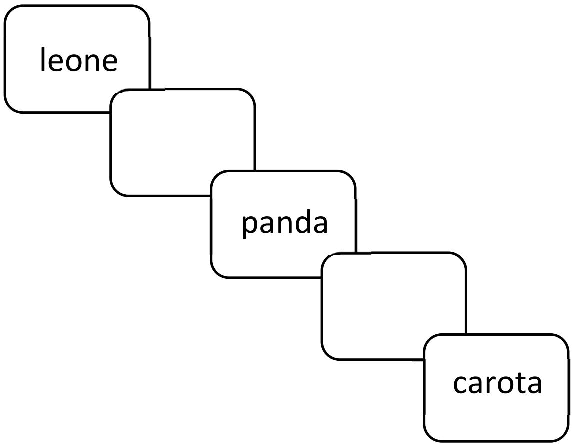

Figure S4. Task-Switching (TS). In some bocks, patients had to respond by pressing the spacebar whenever an animal word starting with the letter “p” appeared (e.g., “panda”). In some block, they had to respond to fruit words starting with the letter “c” (e.g., “carota”, *carrot*). According to alternating-runs paradigm (Rubinstein et al., 2001), in some blocks animal words starting with the letter “p” (or fruit words starting with the letter “c”) represent the target, in other blocks they represent the distracter. The timing of stimuli and blank duration was variable (see the Manuscript). A 38-point Arial font was used and participants were positioned at about 50 cm distance from the screen. The same task was presented with different stimuli across sessions (i.e., geometric symbols, faces, or objects).

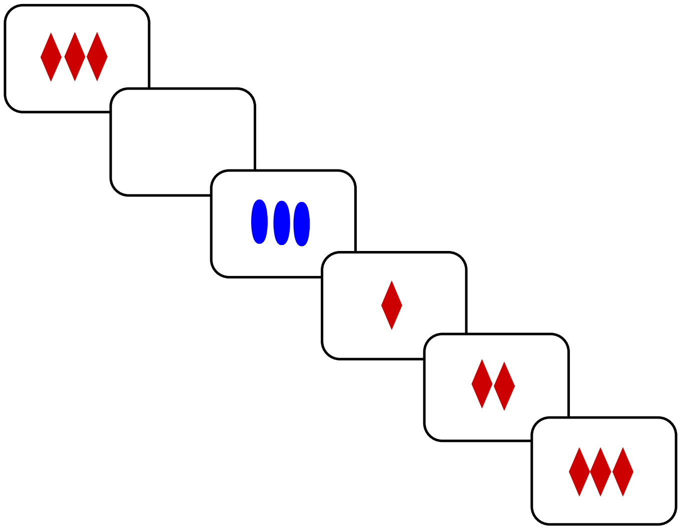

Figure S5. Monitoring task (M). Participants had to remember the target presented at the beginning (in the example, the three-diamonds card), and to detect it, within a series of geometric symbols, by pressing the spacebar. The depicted series is an example of “predictable” series. The timing of stimuli and blank duration was variable (see the Manuscript). Images were contained in a rectangle of 265 × 190 pixels and participants were positioned at about 50 cm distance from the screen. The same task was presented with different stimuli across sessions (i.e., faces, words, or objects).

Authors (country)	N, age	Cognitive deficits at T0	Time since stroke	Software	Trained attention and EFs	Other trained cognitive functions	Training duration (total)
Prokopenko et al., 2013 (Russia) [3]	Intervention: N = 24, Median = 61 yrs (range 60-72); Control: N = 19, Median = 66 yrs (range 60-72)	From mild cognitive impairments to mild dementia (MMSE > 20), without significant speech disorder and aphasia	Within 2 weeks	Home made	Sustained, selective, divided, and switching attention	Spatial cognition	25-35 minutes per day, every day for 14 days (15 hours)
Barker-Collo et al., 2009 (New Zealand) [4]	Intervention: N = 38, M = 70.2 yrs (SD = 15.6); Control: N = 40, M= 67.7 yrs (SD = 15.6)	Attention deficits	Intervention: M = 18.48 days (SD = 11.59); Control: M = 18.58 days (SD = 7.62)	"Attention Process Training"	Sustained, selective, divided, and switching attention	None	1 hour per day, 5 days per week for 4 weeks (up to 30 hours)
De Luca et al., 2018 (Italy) [5]	Intervention: N = 20, M = 43.9 yrs (SD = 16.6); Control: N = 15, M = 42.1 yrs (SD = 17.7)	Moderate cognitive impairment (MMSE from 12 to 20)	Intervention: M= 3.1 months (SD = 1); Control: M = 3.1 months (SD = 1)	"Erica"	Sustained, selective, divided, and switching attention; interference control, abstraction, problem solving	Memory, spatial cognition	45 minutes per day, 3 days per week for 8 weeks (about 24 hours)
Yoo et al., 2015 (South Korea) [6]	Intervention: N = 23, M = 53.2 yrs (SD = 8.8); control: N = 23, M= 56.3 yrs (SD = 7.9)	Not specified	Intervention: M = 11.8 months (SD = 7.5); Control: M= 10.7 months (SD = 6.2)	"RehaCom"	Alertness, vigilance, sustained, selective, divided, and switching attention; working memory	Memory, spatial imagination, visual processing, and visuomotor coordination	30 minutes per day, 5 days per week for 5 weeks (about 13 hours)
Cho et al., 2015 (South Korea) [7]	Intervention: N = 12, M = 60 yrs (SD = 4.7); Control: N = 13, M= 63.7 yrs (SD = 6.3)	Patients were chosen from among those who were able to perform all tests and had light cognitive function impairment (MMSE from 18 to 23)	Intervention: M = 5.3 months (SD = 2.3); Control: M = 6 months (SD = 2.2)	"RehaCom"	Alertness, vigilance, sustained, selective, divided, and switching attention	Not reported	30 minutes per day, 5 days per week for 6 weeks (15 hours)

Authors	Cognitive outcomes	Functional scales	T0-T1 delay	Results on cognitive outcomes	Results on functional scales
Prokopenko et al., 2013 (Russia) [3]	MMSE, FAB, CDT, MoCA, Schulte's test	IADL, CGIS, PGIS, HADS, SS-QoL-12	14-16 days	Compared to T0, the intervention group showed improvement in MMSE, FAB, CDT, Schulte's test, and MoCA.<br>Compared to control group, the intervention group showed better performance at T1 in FAB, CDT, and Schulte's test.	Higher patients' satisfaction with the results of treatment in the intervention group (PGIS).
Barker-Collo et al., 2009 (New Zealand) [4]	IVA-CPT, TMT A and TMT-B, PASAT, Bell test	SF-36, CFQ, GHQ-28, MRS	5 weeks and 6 months	Compared to control group, intervention group showed statistically significant improvement in IVA-CPT after 5 weeks and 6 months.	None
De Luca et al., 2018 (Italy) [5]	MMSE, digit span, attentional matrices, RAVLT, BNT, token test, phonemic verbal fluency, semantic verbal fluency, reversal motor learning, ideomotor and constructive praxia, Raven's colored matrices	ADL, IADL, BI, LCF, HRS-A, HRS-D	After 8 weeks	Compared to T0, both groups improved in MMSE, digit span, phonemic verbal fluency, BNT, token test, constructive praxia, and Raven's colored matrices. Moreover, the intervention group improved in attentional matrices, semantic verbal fluency, and ideomotor praxia. The improvement in the intervention group was significantly larger in MMSE, attentional matrices, semantic verbal fluency, and BNT.	Both groups improved in HRS-A and HRS-D (lower scores), with larger differences in the intervention group.
Yoo et al., 2015 (South Korea) [6]	Digit span, visual span, verbal learning, visual learning, visual CPT, auditory CPT, TMT	FIM	After 5 weeks	Compared to T0, the intervention group improved in digit span, visual span, visual learning, visual CPT, and auditory CPT	None
Cho et al., 2015 (South Korea) [7]	Digit span, visual span, visual CPT, auditory controlled CPT	None	After 6 weeks	Compared to T0, the intervention group improved in digit span, visual span, and visual CPT	-

Table S1. The table summarizes past studies that examined the effects of computerized trainings on attention and executive function in patients with stroke. All studies combined the training with an in-patient standard rehabilitation (intervention group), and included a control group who performed the in-patient standard rehabilitation protocol only.

ADL: Activities of Daily Living; BI: Barthel Index; BNT: Boston Naming Test; CDT: Clock Drawing Test; CFQ: Cognitive Failures Questionnaire; CGIS: Clinical Global Impressions Scale; CPT: Continuous Performance Test; FAB: Frontal Assessment Battery; FIM: Functional Independence Measure; GHQ-28: General Health Questionnaire-28; HADS: Hospital Anxiety and Depression Scale; HRS-A: Hamilton Rating Scale for Anxiety; HRS-D: Hamilton Rating Scale for Depression; IADL: Instrumental Activities of Daily Living; IVA-CPT: Integrated Visual and Auditory Continuous Performance Test; LCF: Levels of

Cognitive Functioning; M: mean; MMSE: Mini Mental State Examination; MoCA: Montreal Cognitive Assessment; MRS: modified Rankin scale; PASAT: Paced Auditory Serial Addition Test; PGIS: Patient Global Impression Scale; RAVLT: Rey Auditory Verbal Learning Test; SD: standard deviation; SF-36: 36-Item Short Form Health Survey; SS-QoL-12: Short Version of the Stroke-Specific Quality of Life Scale; T0: test before intervention (baseline); T1: test after intervention; TMT: Trail Making Test; WMS: Wechsler Memory Scale; yrs = years.

References

1. Barbour, V.; Bhui, K.; Chescheir, N.; Clavien, P.A.; Diener, M.K.; Glasziou, P.; Golub, R.M.; Grimshaw, J.M.; Groves, T.; Hopewell, S.; et al. CONSORT Statement for randomized Trials of nonpharmacologic treatments: A 2017 update and a CONSORT extension for nonpharmacologic Trial Abstracts. *Ann. Intern. Med.* **2017**, doi:10.7326/M17-0046.
2. Rubinstein, J.S.; Meyer, D.E.; Evans, J.E. Executive control of cognitive processes in task switching. *J. Exp. Psychol. Hum. Percept. Perform.* **2001**, *27*, 763–797, doi:10.1037/0096-1523.27.4.763.
3. Prokopenko, S. V.; Mozheyko, E.Y.; Petrova, M.M.; Koryagina, T.D.; Kaskaeva, D.S.; Chernykh, T. V.; Shvetzova, I.N.; Bezdenezhnih, A.F. Correction of post-stroke cognitive impairments using computer programs. *J. Neurol. Sci.* **2013**, *325*, 148–153, doi:10.1016/j.jns.2012.12.024.
4. Barker-Collo, S.L.; Feigin, V.L.; Lawes, C.M.M.; Parag, V.; Senior, H.; Rodgers, A. Reducing attention deficits after stroke using attention process training: A randomized controlled trial. *Stroke* **2009**, *40*, 3293–3298, doi:10.1161/STROKEAHA.109.558239.
5. De Luca, R.; Leonardi, S.; Spadaro, L.; Russo, M.; Aragona, B.; Torrisi, M.; Maggio, M.G.; Bramanti, A.; Naro, A.; De Cola, M.C.; et al. Improving Cognitive Function in Patients with Stroke: Can Computerized Training Be the Future? *J. Stroke Cerebrovasc. Dis.* **2018**, *27*, 1055–1060, doi:10.1016/j.jstrokecerebrovasdis.2017.11.008.
6. Yoo, C.; Yong, M.H.; Chung, J.; Yang, Y. Effect of computerized cognitive rehabilitation program on cognitive function and activities of living in stroke patients. *J. Phys. Ther. Sci.* **2015**, *27*, 2487–2489, doi:10.1589/jpts.27.2487.
7. Cho, H.Y.; Kim, K.T.; Jung, J.H. Effects of computer assisted cognitive rehabilitation on brain wave, memory and attention of stroke patients: A randomized control trial. *J. Phys. Ther. Sci.* **2015**, *27*, 1029–1032, doi:10.1589/jpts.27.1029.
